# Harvey Cushing and the Secretion of the Posterior Pituitary into the C.S.F.

**Published:** 1988-02

**Authors:** T Z Aziz

**Affiliations:** Frenchay Hospital


### Harvey Cushing and the Secretion of the Posterior Pituitary Into the Cerebro Spinal Fluid

**T Z Aziz, BSc. MB, BS, SHO. Frenchay Hospital.**

Harvey Cushing is most commonly remembered by those in medical practice for his description of Cushing's disease caused by hyper secretion of ACTH by pituitary micro adenomas. This however, was a contribution made very much towards the end of a phenomenal career in which he made important discoveries in several branches of medicine. In neurosurgery, some may argue that McEwen of Glasgow or Horsley of London rather than Cushing was the founder of the speciality. However Harvey Cushing was the first full time neurosurgeon and it was his work in the early days of the speciality that made it safe. Indeed, when he started in neurosurgery in 1899 the reported mortality for neurosurgical procedures was close to a 100% but by 1915 when he published a paper "concerning the results of operations for brain tumours", his mortality rate on average was 8.4%; higher in some procedures compared to others. At the same time the reported mortality of Kuttner of Breslau was 45%, that of Krause of Berlin was 50%, that of Eiselburg of Vienna was 38% and Horsley's was estimated at 38%. Meningitis claimed the lives of 10.5% of post operative patients in Vienna; 11.7% of Horsley's patients; but Cushing reported only one such case in his series. (1).

In making neurosurgery safe, Cushing introduced the scalp tourniquet (now obsolete) to reduce haemorrhage, silver clips to clamp bleeding vessels without causing tissue trauma by suture ligation, and diathermy to reduce operative bleeding.

In anaesthetics, Cushing and Codman introduced the ether chart in 1895 in which a patient's pulse and respiratory rate were continuously plotted during an anaesthetic and in 1900 he introduced the Riva Rocci sphygmomanometer with its inflatable arm cuff into anaesthesia to monitor the blood pressure (2).

Harvey Cushing also prided himself on the fact that he was a member of the American Physiological Society and of his contributions to that science. His earliest venture into it was with Kocher in 1901 when after two months of laboratory work, he described the "Cushing reflex" in which the pulse rate falls and the blood prssure rises as intra cranial pressure increases. In 1909 his work on stimulation of the post central gyrus in conscious patients established its role as a primary sensory receptive area (2). His work described above is well recognised but what has for me been his most fascinating physiological work was that on secretion of the posterior pituitary into the cerebro spinal fluid. My interest in it stems from the fact that it was widely derided by other physiologists of the time, it took about seventy years to prove true, and his role in its discovery seems to have been largely forgotten.

Harvey Cushing's belief that the posterior pituitary secreted into the CSF was conjectural, based on tenuous anatomical findings. In 1908 P.T. Herring put forward the suggestion that the colloid masses in the pars intermedia represented secretory products of the investing epithelial cells and that these found their way through the tissue interstices of the posterior lobe to be ultimately discharged between the ependymal cells into the cavity of the third ventricle.

Cushing and Goetsch in 1910 (3) reported their work in animal preparations supporting the hypothesis. In six animal preparations, they were able to demontrate an increase in the colloid seen and also an increase in hyaline bodies (secretory granules) in the posterior pituitary itself. These two structures could always be demonstrated in close histological relation to the ependymal cells of the third ventricle.

Dogs were studied:

(1) after extirpation of other ductless glands—thyroid and pancreas.
(2) after mechanical injury—injection of india ink into the pituitary.
(3) after partial hypophysectomy.
(4) after total hypophysectomy in which remnants of the stalk were left behind.
(5) after partial obstruction of the pituitary stalk with a silver clip; and
(6) after transplanting the pituitary to another part of the brain. However, the colloid when extracted was inactive when injected into experimental animals and it could not be ascertained whether the colloid and the hyaline bodies in the posterior pituitary itself were related.

Nevertheless, he went on to postulate that perhaps the posterior pituitary could activate the colloid substance in some manner before it was secreted into the third ventricle.

Prior to this study by Cushing, Howell and Schafer had studied the effects of extracts of the posterior pituitary and had found that subcutaneous or intravenous injections of it had such effects as increasing arterial pressure, causing diuresis, stimulation of smooth muscle as seen in the uterus, bladder and intestine and dilation of the pupil of the isolated frogs eye.

With this knowledge of what posterior pituitary extracts could do in the experimental animal, a bioassay, Cushing and Goetsch went on to study the activity in CSF.

They began their studies in June 1910. An infant with congenital obstructive hydrocephalus presented to them and whilst deciding on a definitive form of treatment, a numbert of ventricular and lumbar punctures were made and large amounts (300–600 mls) of CSF obtained. The fluid was concentrated over a water bath to one sixth of its volume and injected into the external jugular vein of an anaesthetised dog and an unanaesthetised rabbit. In both preparations, after an initial short-lived fall in blood pressure, there was an enduring rise in the BP, a fall in pulse rate , amplification of the pulse and rise in the respiratory rate. These reactions were also those obtained on injecting posterior pituitary extracts.

They went on to use a rabbit as the bioassay of choice and to avoid artefacts caused by injecting large volumes of fluid intravenously, a standard degree of concentration was adopted; 50 mls of CSF concentrated to 2.5 mls. In the quiet and partly eviscerated rabbit, marked contractions of the intestine, bladder and uterus were noted and in one case, injection into a branch of the mesenteric artery caused vigorous contractions of the supplied intestinal loops. A further eight patients were studied, two had obstructive hydrocephalus secondary to tumour, six patients had either epilepsy, acromegaly, Frolich's syndrome or recent trauma.

All fluids tested were able to raise the BP after an initial fall, slow and amplify the pulse, cause diuresis, contract the musculature of intestine bladder and uterus and dilate the pupil of the isolated frog eye. However, it was noted that cases with obstructive hydrocephalus were most potent in their effects. This was explained by a continued posterior lobe secretion into ventricles with no possibility of escape into the general circulation. Traumatic and epileptic cases gave the least potent CSF samples.

Cushing went on to suggest that the CSF could be used as a source of posterior lobe hormones to be extracted and remarkably suggested that there may be two hormones a pressor and a depressor substance, the latter causing dilation of the frogs eye.

In the last 20 years the truth of Harvey Cushings supposition has been borne out. The posterior pituitary does secrete two substances, vasopressin and oxytocin; both are fully characterised and synthesised. Their actions were until very recently believed to be exerted only by secretion into the blood stream.

Nevertheless, in the last decade evidence has confirmed the presence of posterior pituitary hormones in the CSF, and neurosecretory path ways for vasopressin secretion directly into the third ventricle have been identified. Harvey Cushing's choice of patients has also been fully supported in that patients with an increased intra cranial pressure are those in which vasopressin is most elevated (4–8). The role for this CSF pathway is still to be fully understood but there is evidence that it may be of importance in learning mechanisms, brain water permeability and in the regulation of peripheral levels. (9).

I wondered why Cushing's work in this area remained so totally ignored. The answer lies in the fact that it was so ahead of its time that confirmation required the passage of 60 years and the development of radio immunoassays. The scepticism of his contemporaries also did not help; indeed John Fulton in Cushing's biography was to say "In the papers on posterior pituitary secretion, he had been led astray, but despite an impressing array of evidence to the contrary, he never really admitted that he was wrong; in addition, he unfortunately caused a number of his junior associates to waste valuable time and effort in attempting to establish his original contention". (2) Harvey Cushing was a man with an obsession for priority; I hope that this short review will point people in the direction of his work in this field and also to realise that reviewing papers long in the past can contribute to work in these modern times.

### REFERENCES

1. THOMPSON, E. H. (1950) Harvey Cushing, Surgeon, Author Artist. New York, Henry Schuman.
2. FULTON, S. F. (1946)—Harvey Cushing, A Biography. Oxford. Blackwell Scientific Publications.
3. CUSHING, H. and GOETSCH, E. (1910)—"Concerning the secretion of the infundibular lobe of the pituitary body and its presence in the cerebro spinal fluid: *AM.H.Physiol.* 1 Nov. **27**, p.60–86.
4. SORENSEN, P. S., HAMMER, M. and FLEMMING, G. (1982)—"Cerebro spinal fluid vasopressin in benign intra cranial hypertension". *Neurology* (NY); **32**: 1255–9.
5. SORENSEN, P. S. HAMMER, M. GJERRIS, F. *et al* (1985)—"24 hour cerebro spinal fluid levels of vasopressin in hydrocephalic patients". Regulatory peptides, **10**, 115–126.
6. SORENSEN, P. S. HAMMER, W. VORSTRUP, S. *et al* (1983)—"CSF and plasma vasopressin concentrations in dementia". *J.Neurol. Neurosurg.Psychiat.* **46**, 911–916.
7. SORENSEN, P. S. GJERRIS, F. and HAMMER, M. (1984)—"Cerebro spinal fluid vasopressin and increased intra cranial pressure *Ann. of Neurol.* May **15**(5), p. 435–40.
8. BERGLAND, R. M. PAGE, R. B. (1978)—Can the pituitary secrete directly to the brain? (affirmative anatomical evidence) *Endocrinology* **102**(5), p. 1325–38.
9. DE WEID, D. (1980) Hormonal influences on motivation learning, Memory and psychosis. Chap 21, Neuro-endocrinology. Edited by D. T. Krieger, J. C. Hughes. Massachusets, Sinauer Assoc.

